# Bi_2_MoO_6_ Embedded in 3D Porous N,O-Doped Carbon Nanosheets for Photocatalytic CO_2_ Reduction

**DOI:** 10.3390/nano13091569

**Published:** 2023-05-06

**Authors:** Xue Bai, Lang He, Wenyuan Zhang, Fei Lv, Yayun Zheng, Xirui Kong, Du Wang, Yan Zhao

**Affiliations:** 1 The Institute of Technological Sciences, Wuhan University, Wuhan 430072, China; xuebai@whu.edu.cn (X.B.); 2021206520034@whu.edu.cn (F.L.); zhengyayun@whu.edu.cn (Y.Z.); urumqi-racy@whu.edu.cn (X.K.); 2 School of Chemistry and Chemical Engineering, Harbin Institute of Technology, Harbin 150080, China; wyzhang@hit.edu.cn

**Keywords:** CO_2_RR, N,O-doped carbon, photocatalysis, Bi_2_MoO_6_

## Abstract

Artificial photosynthesis is promising to convert solar energy and CO_2_ into valuable chemicals, and to alleviate the problems of the greenhouse effect and the climate change crisis. Here, we fabricated a novel photocatalyst by directly growing Bi_2_MoO_6_ nanosheets on three-dimensional (3D) N,O-doped carbon (NO-C). Scanning electron microscopy (SEM) and transmission electron microscopy (TEM) show that the designed photocatalyst ensured the close contact between Bi_2_MoO_6_ and NO-C, and reduced the stacking of the NO-C layers to provide abundant channels for the diffusion of CO_2_, while NO-C can allow for fast electron transfer. The charge transfer in this composite was determined to follow a step-scheme mechanism, which not only facilitates the separation of charge carriers but also retains a strong redox capability. Benefiting from this unique 3D structure and the synergistic effect, BMO/NO-C showed excellent performance in photocatalytic CO_2_ reductions. The yields of the best BMO/NO-C catalysts for CH_4_ and CO were 9.14 and 14.49 μmol g^−1^ h^−1^, respectively. This work provides new insights into constructing step-scheme photocatalytic systems with the 3D nanostructures.

## 1. Introduction

It is well known that we have advanced from an industrialized era to an information era. One of the grand challenges in this new era is to reduce the emission of greenhouse gases, which are mainly due to the excessive consumption of fossil fuels [1,2,3]. Climate change has posed a threat to the ecosystem that human beings, caused by the gradual increase of the concentration of CO_2_ [4,5], which arises from industrial emissions and excessive burning of fossil fuels [6,7]. To mitigate the increasing concentration of CO_2_ in the atmosphere, one effective strategy is to photocatalytically convert CO_2_ to valuable solar fuels, such as hydrocarbons [8,9,10], which provide a viable solution for future energy storage and supply [11].

Carbon materials possess considerable microstructural and electrical properties [12], and are facile to synthesize, making them one of the excellent substrate materials for a wide range of applications [13,14]. Carbon materials serve as an excellent substrate to support exotic catalysts due to their thin layer structure and excellent electron transport ability [15], and N atom-doped carbon materials not only prevent catalyst aggregation but also provide higher conductivity and expose more active sites [16,17].

Unfortunately, the rapid recombination of photogenerated electrons and holes limits the applications of graphitic materials for the photocatalysis [18,19]. Some methods have been developed to overcome this problem, including doping with metallic and nonmetallic elements, mixing with metal oxides or metal sulfides, heterostructure constructions, and morphology modification [16,17,18,19]. Moreover, Liu et al. proposed that the energy band structure of the material is tuned by the construction of heterojunctions [19,20]. Christoffer et al. employ ab initio molecular dynamics (AIMD) simulations to demonstrate that the loaded Bi-containing material not only maintains and promotes the topological insulating properties but also improves the original band gap [21].

**Scheme 1:**
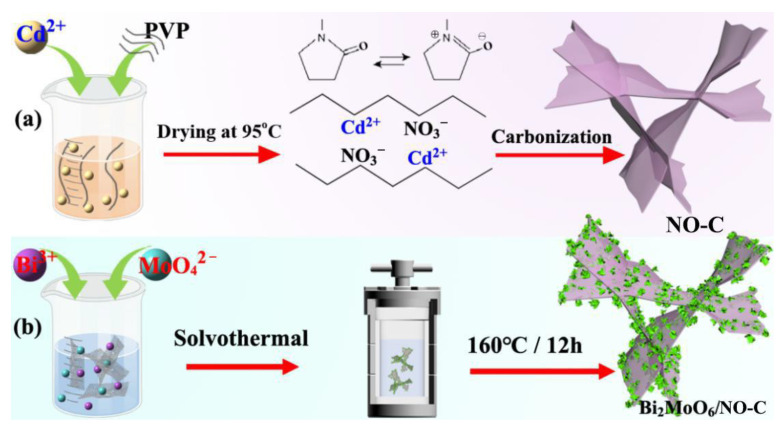
Schematic illustration of the formation of nanocomposite, (**a**) NO-C; (**b**) BMO/NO-C.

Bismuth molybdate (Bi_2_MoO_6_) as a typical n-type semiconductor, which possesses a remarkable layered structure and a proper band gap (2.5~2.7 eV) [22,23], is often used to construct heterostructures with other semiconductors to achieve the promotion of photoinduced electron-hole pair separation and to improve the photocatalytic performance of heterostructured materials. In this work, a CO_2_RR photocatalyst was fabricated by uniformly dispersing Bi_2_MoO_6_ (BMO) nanosheets on 3D N-doped graphitic carbon (NO-C), using a solvent thermochemical synthesis method, and the design is shown in Scheme 1. The direct growth of Bi_2_MoO_6_ nanosheets on 3D NO-C effectively prevents the restacking of NO-Cs. Furthermore, the unique 3D contact and coupling are favorable for carrier separation, and provide effective channels for rapid electron transfer, further increasing the charge transfer rate of the material under photoexcitation. The optimized BMO/NO-C-7 catalyst gives good CH_4_ yield, and high CO selectivity, with strong CO_2_ reduction efficiency and stability after 35 h.

## 2. Experimental Section

Supporting information gives the details of experiments, materials characterizations, electrochemical tests, and DFT calculations.

### 2.1. Preparation of the Ultra-Thin NO-C Nanosheets

The method used to fabricate the ultra-thin 3D NO-C nanosheets was based on a previous report [23,24]. Firstly, the PVP-Cd^2+^ precursor was formed by dissolving 1.0 g of PVP in 30 mL of DI water and adding 1.5 g of Cd(NO_3_)_2_·6H_2_O. The mixture was stirred at room temperature to ensure it was homogeneous. The solution was then dried in an oven at 95 °C to form the precursor. Subsequently, the precursor was heated up to 1000 °C in a tube furnace that was protected by an N_2_ atmosphere, with a heating rate of 5 °C min^−1^ (The N_2_ flow rate is 80~100 mL·min^−1^). Finally, the ultra-thin 3D NO-C nanosheets were obtained by carbonization at 1000 °C for 1 h.

### 2.2. Preparation of BMO/NO-C Composites

The synthesis of Bi_2_MoO_6_/NO-C (BMO/NO-C) was accomplished by a solvent thermochemical method [25]. Initially, a clear and transparent solution was formed by mixing Bi(NO_3_)_2_·5H_2_O (0.6 mmol) and Na_2_MoO_4_·H_2_O (0.3 mmol) in ethylene glycol (5 mL). After stirring for an hour, 30 mL of ethanol was added to the mixture, and the NO-C (50, 70, 90 mg) was incorporated and stirred for an additional hour. Finally, the mixed solution was heated for 12 h in a sealed autoclave at 160 °C. The resulting products were washed repeatedly with ethanol and allowed to dry. The products were named BMO/NO-C-5, BMO/NO-C-7, and BMO/NO-C-9, according to the quantity of NO-C that was added to the mixture.

## 3. Results and Discussions

### 3.1. Preparation and Material Characterization

The pure ultra-thin 3D NO-C sheets were prepared by a simple polymer thermal treatment method. Scheme 1a shows that, after Cd(NO_3_)_2_·4H_2_O was added to the aqueous PVP solution, the Cd^2+^ ions can form strong interactions with the amide groups in the PVP chains, and PVP becomes a stabilizer of the dissolved Cd^2+^ ions. When the water in the solution is removed by drying at 95 °C, Cd^2+^ ions are uniformly distributed in the networks of the amide and methylene groups of the pyrrolidine ring of PVP. During the carbonization stage, PVP decomposes to form NO-C, and some CdO nanoparticles are formed. However, at a high temperature of 1000 °C, CdO is further reduced to Cd metal vapor. With the flow of N_2_, the Cd vapor was removed from the NO-C nanosheets. Scheme 1b shows the fabrication of BMO/NO-C composites. Bi(NO_3_)_2_·5H_2_O and Na_2_MoO_4_·2H_2_O form the Bi^3+^ and MoO_4_^2−^ ions after completely dissolved in ethylene glycol and ethanol. During the solvothermal stage, BMO nanosheets have grown on the 3D NO-C substrates to form the BMO/NO-C catalysts.

Figure S1a gives the TGA-DTG analysis of the Cd^2+^-PVP precursor, and the TGA curve exhibits three different stages of decomposition. The initial mass loss before 220 °C is due to the loss of the water residue (~13%). The DTG peak that occurs at 335 °C is attributed to the decomposition of the NO_3_^−^ anions. The second stage of decomposition occurs between 220–440 °C and corresponds to a ~67% mass loss, indicating that most of the Cd^2+^-PVP precursor has been decomposed. The mild mass loss after 800 °C can be attributed to the vaporization of some Cd species.

Figure S1b shows a typical SEM image of the NO-C, which exhibits a wrinkled, fluffy, and disordered graphene-like morphology [26,27]. Figure S1c shows the X-ray diffraction (XRD) patterns of NO-C. The diffraction peaks of 22.7 and 34.2 in the XRD pattern correspond to the lattice planes (002) and (103) of graphitic carbon (JCPDS, No. 50-0926) [28]. Figure S1d presents the Raman spectrum of NO-C, which exhibits two characteristic peaks at 1340 cm^−1^ (D-band) and 1590 cm^−1^ (G-band) [29,30].

**Figure 1:**
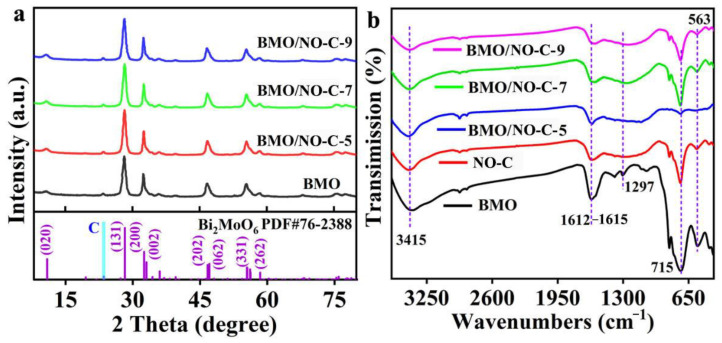
(**a**) XRD diffraction patterns of BMO/NO-C-5, BMO/NO-C-7, BMO/NO-C-9, and BMO (The standard JCPDS cards of Bi_2_MoO_6_ are shown under the XRD pattern). (**b**) FT-IR of BMO/NO-C-5, BMO/NO-C-7, BMO/NO-C-9, NO-C, and BMO.

Figure 1a exhibits the XRD patterns of pure BMO and the BMO/NO-C catalysts. The strong diffraction peaks at 2θ = 28.1°, 32.2°, 46.6°, and 55.3° are attributed to the (131), (200), (062), and (331) crystal planes of Bi_2_MoO_6_ (JCPDS, No. 76-2388), respectively, and no impurity peaks detected. The FT-IR spectra in Figure 1b further confirmed that the composites were successfully prepared. The infrared peak located at 563 cm^−1^ corresponds to the stretching vibration of Bi–O, and a strong infrared peak located at 715 cm^−1^ corresponds to the asymmetric bending vibration mode of MoO_6_ [31]. In BMO, two vibrational peaks at 1615 cm^−1^ and 3415 cm^−1^ can be assigned to chemisorbed water molecules and O–H stretching [32,33]. The peak at 3415 cm^−1^, indexed to a hydroxyl group (–OH) [32], originates from the residual water in the catalysts.

Figure S2 presents the specific surface area and pore size distributions of BMO and the BMO/NO-C catalysts revealed by nitrogen adsorption–desorption isotherms, which shows that the BMO/NO-C catalysts exhibit IV-type adsorption isotherms and a type-H3 hysteresis loop. The specific surface areas (S_BET_) are 47.72, 50.18, 51.38, and 49.69 cm^2^/g for BMO, BMO/NO-C-5, BMO/NO-C-7, and BMO/NO-C-9, respectively. It is worth noting that the BMO/NO-C-7 catalyst gives the highest S_BET_, which can be attributed to the uniform distribution of BMO nanosheets. As more NO-C was added to the reaction system, the disordered assembly between some of the BMO nanosheets led to aggregation, which instead reduced the surface pore space and the specific surface area [33,34,35]. The inset in Figure S2 shows that the BMO/NO-C catalysts exhibit a typical mesoporous structure, which provides not only abundant channels for the diffusion of CO_2_ gas on the surface and inside the catalysts but also contributes many active sites for the adsorption and reduction of CO_2_, which is very beneficial for improving the photocatalytic CO_2_RR performance [36,37,38].

**Figure 2:**
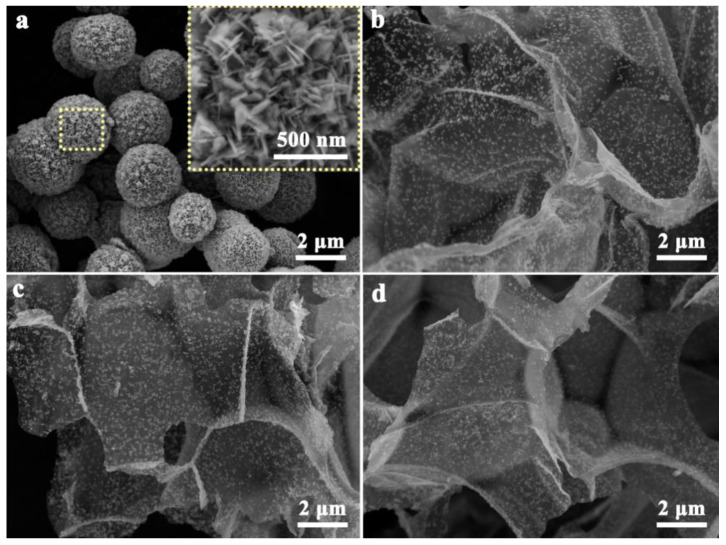
SEM images of BMO (**a**), BMO/NO-C-5 (**b**), BMO/NO-C-7 (**c**), and BMO/NO-C-9 (**d**).

**Figure 3:**
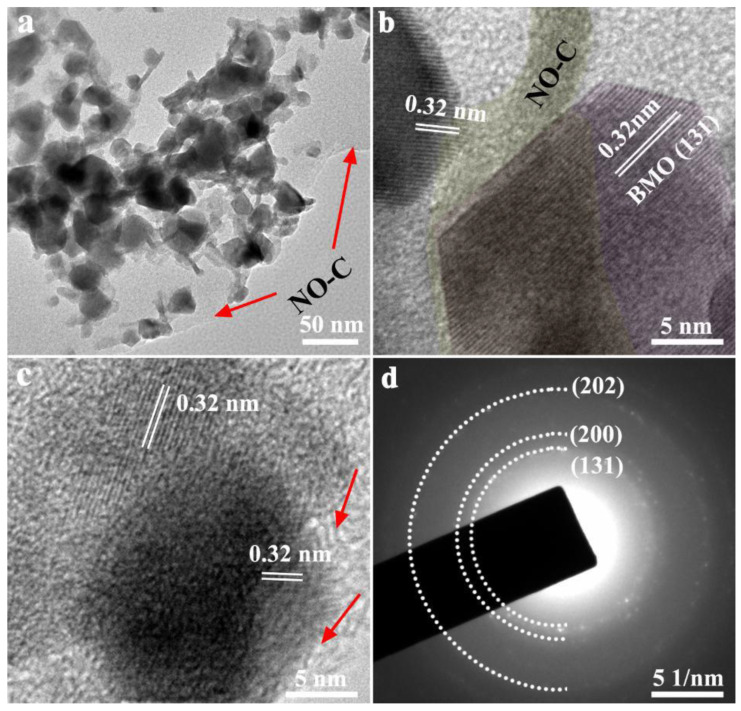
(**a**) TEM images of BMO/NO-C-7, (**b**) and (**c**) HRTEM images of BMO/NO-C-7, (**d**) SAED pattern of BMO/NO-C-7.

The microstructure and morphology of the catalysts were characterized by using SEM. The inset of Figure 2a has shown that Bi_2_MoO_6_ is spherical and composed of a large number of nanosheets. In the BMO/NO-C catalysts (Figure 2b–d), BMO nanosheets are embedded in the NO-C substrates, which is confirmed by the TEM and high-resolution TEM (HRTEM) results in Figure 3. As shown in Figure 3a, BMO nanosheets are uniformly distributed on ultra-thin N-doped graphitic carbon networks. The HRTEM in Figure 3b,c gives lattice distances of 0.32 nm of the embedded BMO, which corresponds to the (131) crystalline fringe. The HRTEM images reveal that the heterojunctions and defects are formed at the interfaces of BMO and NO-C, providing active sites for the adsorption and reduction of CO_2_ [39]. Atomic force microscopy (AFM) images further confirmed that the carbon nanosheets are ultrathin (Figure S4a,b). The thickness of the completely randomly measured NO-C nanosheets ranged from 1.4~2 nm (Figure S4a,b). The formation of ultra-thin, porous structures can expose abundant active sites favorable for gas diffusion on the material surface, and the use of ultrathin nanosheets can enhance the photocatalytic activity, thus reducing the charge migration distance and decreasing the recombination rate of photogenerated carriers [40].

The selected area electron diffraction (SAED) of BMO/NO-C-7 is shown in Figure 3d. The white dashed lines are the Bi_2_MoO_6_ (131), (200), and (202) crystal planes. The SAED results are consistent with the XRD ones.

**Figure 4:**
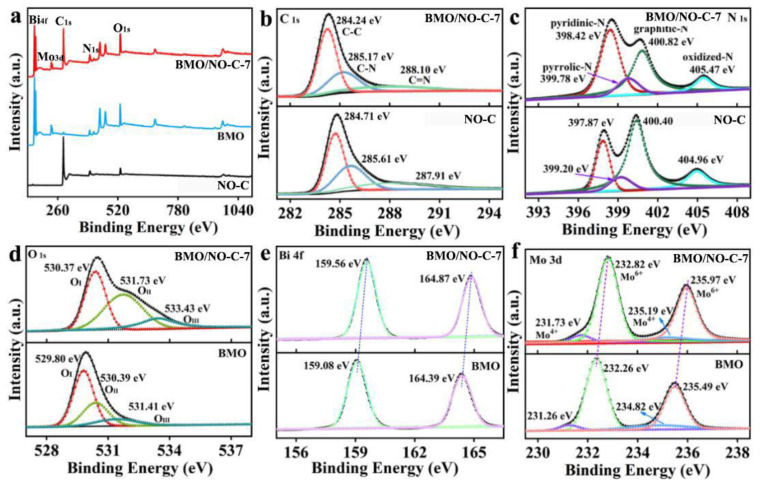
(**a**) XPS survey spectra of BMO, NO-C, and BMO/NO-C-7. High-resolution XPS spectra of (**b**) C 1s, (**c**) N 1s, (**d**) O 1s (**e**) Bi 4f, and (**f**) Mo 3d of the BMO and BMO/NO-C-7.

X-ray photoelectron spectroscopy (XPS) was employed to determine the chemical state of the catalysts. Figure 4a displays the survey XPS spectra of NO-C, BMO, and BMO/NO-C-7. The XPS peaks for C 1s can be seen in NO-C, whereas the XPS peaks for Bi 4f, Mo 3d, and O 1s peaks are revealed in the BMO XPS spectrum. The BMO/NO-C-7 XPS exhibits the combined peaks of NO-C and BMO [41]. Figure 4b is the XPS spectra of C 1s for the pure NO-C and BMO/NO-C-7 composites. The peaks at 284.24 (284.71), 285.17 (285.61), and 288.10 (287.91) eV are identified as the sp^2^ C-C [7,40], C-N [42], and N-C=N [27] bonds in BMO/NO-C-7 (NO-C). The N 1s XPS spectrum of BMO/NO-C-7 (Figure 4c) shows four peaks at 398.42, 399.78, 400.82, and 405.47 eV, corresponding to the pyridinic-N [43], pyrrolic-N [44], graphitic-N [45], and oxidized-N [46], respectively. The O 1s XPS spectra (Figure 4d) exhibit three main peaks at 530.37, 531.73, and 533.43 eV, which are indexed to the lattice oxygen (O_Ⅰ_) [47], defect oxygen (O_Ⅱ_) [48,49], and chemisorbed oxygen (O_Ⅲ_) [49,50]. Figure 4e presents the Bi 4f XPS spectra of BMO/NO-C-7, and the two main peaks at 159.56 and 164.87 eV can be identified as Bi 4f_7/2_ and Bi 4f_5/2_ of Bi^3+^, respectively [51]. Figure 4f exhibits the Mo 3d XPS spectra, in which two major peaks at binding energies of 231.73 and 235.19 eV are indexed to Mo 3d_3/2_ and Mo 3d_5/2_ of Mo^4+^ [52], whereas the two remaining peaks at 232.82 and 235.97 eV arise from Mo 3d_3/2_ and Mo 3d_5/2_ of Mo^6+^ [53]. As compared to pure BMO, the XPS peaks for Bi and Mo in BMO/NO-C-7 all shifted to higher binding energies, indicating that some electrons transfer at the interface of BMO and NO-C.

**Figure 5:**
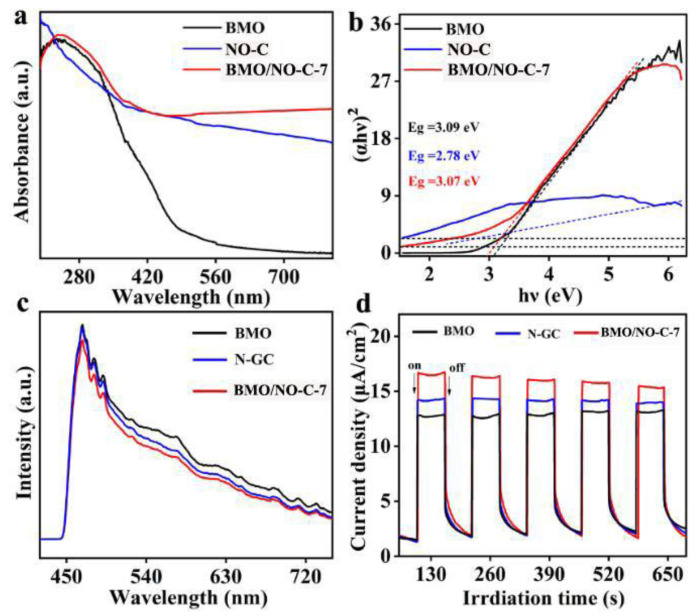
BMO/NO-C-7, NO-C, and BMO of (**a**) UV–vis diffuse reflectance spectra, and (**b**) Plots of (*ahν*)^2^ versus energy (*hν*). (**c**) PL spectra of the BMO, NO-C, and BNO/NO-C. (**d**) transient photocurrent response of BMO, NO-C, and BMO/NO-C-7.

UV–Vis diffuse spectra were employed to investigate the optical absorption properties of the catalysts. Figure 5a shows that BMO/NO-C-7 exhibits superior absorption in the UV and visible region as compared to pure BMO or NO-C, indicating higher photocatalytic activity. In addition, the band gap (*E_g_*) of the catalysts has been calculated with the Kubelka–Munk formula: *(ahν)^2/n^ = A (hν − E_g_)* (α: absorption coefficient, *hν*: photon energy, *A*: a proportionality constant, *n* = 1 is for the direct band gap and *n* = 4 is for indirect) [54]. The calculated direct band gaps for BMO, NO-C, and BMO/NO-C-7 are 3.09, 2.78, and 3.07 eV, respectively (Figure 5b). The charge separation and transfer properties of the catalysts were further explored by using photoluminescence (PL) spectroscopy. At the excitation wavelength of 375 nm (Figure 5c), the pure BMO exhibit a strong PL intensity, and the BMO/NGC-7 catalyst gives the weakest intensity. It is well known that low PL intensity represents a high separation efficiency of the photogenerated carriers, which indicates an effective charge separation and transfer between BMO and NO-C [55]. The transient photocurrent responses of the BMO, NO-C, and BMO/NO-C-7 catalysts were recorded in Figure 5d, which shows that the photocurrent of the BMO/NO-C-7 catalyst is significantly higher than that of BMO or NO-C. BMO/NO-C-7 exhibits the highest current density, indicating a much higher separation efficiency of the light-generated charge carriers [56].

**Figure 6:**
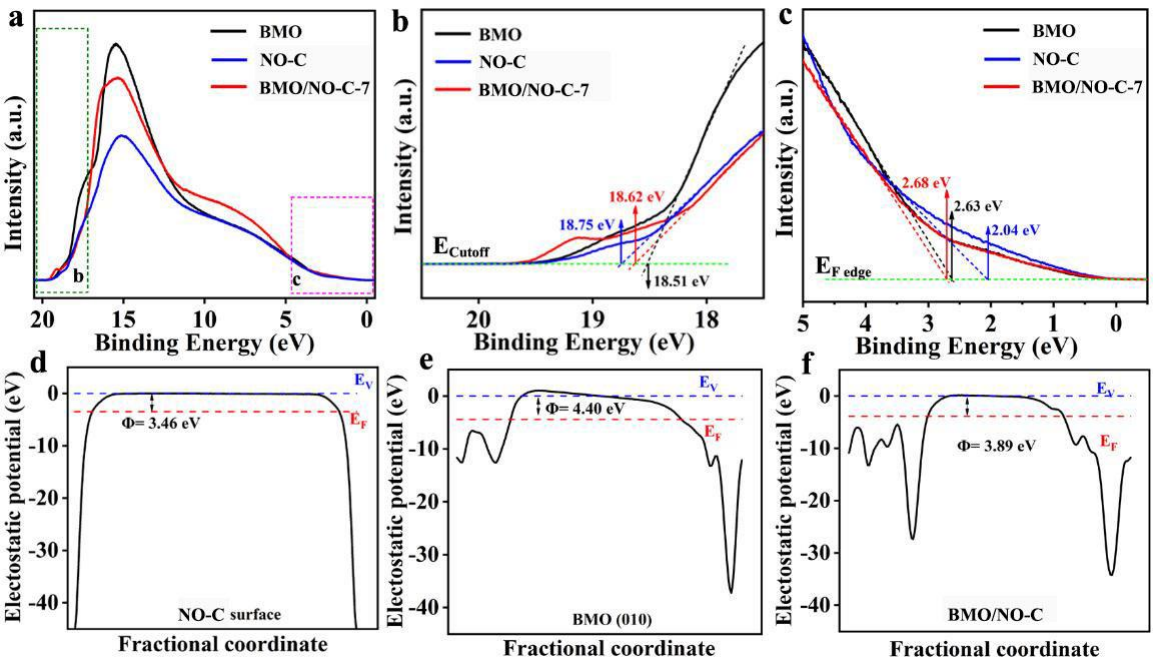
(**a**) UPS spectra of the BMO, NO-C, and BMO/NO-C films, (**b**) cutoff energy (the dashed lines mark the baseline and the tangents of the curve) and (**c**) the intersections of the tangents with the baseline give the fermi (*E_F_*) edges. Calculated electrostatic potentials (Φ) of (**d**) NO-C and (**e**) Bi_2_MoO_6_. (**f**) BMO/NO-C heterojunction slab model. The Fermi level is marked with a dashed red line, and the vacuum level is marked with a dashed blue line.

Ultraviolet photoelectron spectroscopy (UPS) was employed to measure the valence band (VB) and the conduction band (CB) of the BMO, NO-C, and BMO/NO-C composite, shown in Figure 6a. The *E_cutoff_* energies of BMO, NO-C, and BMO/NO-C are 18.51, 18.75, and 18.62 eV, respectively (Figure 6b), while the *E_F-edge_* energies for three catalysts were 2.63, 2.04, and 2.68 eV, respectively (Figure 6c). Based on the equation *E_VB_* = *−IP* (ionization potential) = −(*hν − (E_cutoff_ − E_F-edge_*)), where *hν* is the He I photon source energy (21.21 eV), the valence band energies (*E_VB_*) of BMO, NO-C, and BMO/NO-C are −5.33, −4.50, and −5.27 eV, respectively [57,58]. According to the equation *E_CB_ = E_VB_ + E_g_* (the *E_g_* energies were taken from Figure 5), the conduction band energies (*E_CB_*) for BMO, NO-C, and BMO/NO-C are −2.24, −1.72 and −2.20 eV, respectively [59]. Significantly, the Fermi level of BMO is lower than that of NO-C, indicating that electrons are transferred from NO-C to BMO and form heterojunctions [60,61,62]. The DFT calculated work functions (Φ) of NO-C and BMO are 3.46 eV and 4.40 eV, respectively (Figure 6d,e), which is consistent with the experimental results that the *E_F_* of NO-C is higher than that of BMO. When BMO and NO-C are in contact to form a composite, electrons spontaneously transfer from NO-C to BMO until reaching equilibrium [63]. Φ of the BMO/NO-C heterojunction is 3.88 eV (Figure 6f), which is between the work functions of BMO and NO-C. In the BMO/NO-C composite, the BMO accumulates some negative charge, whereas the NO-C layer accumulates some positive charges, which is confirmed by the differential charge density analysis of DFT calculations (Figure S3d). The accumulated positive charges on NO-C and negative charges on BMO induce an internal electric field from NO-C to BMO. It is worth mentioning that the internal electric field at the interface exerts electrostatic forces on the photogenerated holes and electrons, forming a heterojunction for photocatalytic carrier separation and diffusion [64,65]. 

### 3.2. Photocatalytic CO_2_ Reduction

**Figure 7:**
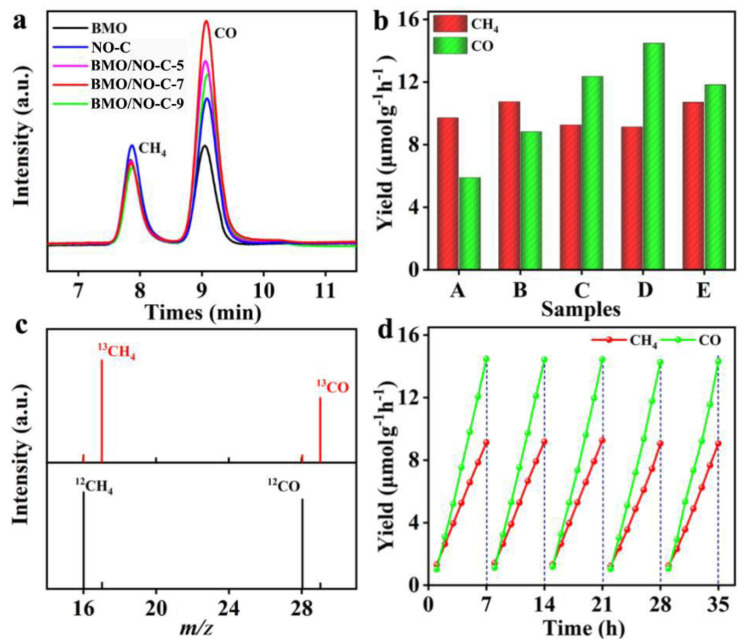
(**a**) The CO_2_ reduction spectrum of all composites accumulated for 1 h of light irradiation. (**b**) Photocatalytic activity of different samples for CO_2_ reduction (A: BMO, B: NO-C, C: BMO/NO-C-5, D: BMO/NO-C-7, E: BMO/NO-C-9). (**c**) The GC-MS patterns of the produced CH_4_ and CO over BMO/NO-C-7 using ^12^CO_2_ and ^13^CO_2_ as the carbon source, respectively. (**d**) The photocatalytic cycle performance of BMO/NO-C-7.

To determine the photocatalytic performance of the BMO/NO-C catalysts, we performed photocatalytic CO_2_ reduction experiments with the Labsolar 6A system from Beijing Perfectlight Technology Co., Ltd., (Beijing, China) which is an all-glass automatic online trace gas analysis system coupled with a 300 W Xenon lamp source (Microsolar300, Figure S4). Figure 7a shows that CH_4_ and CO are the main products of photocatalytic CO_2_ reduction, and Figure 7b compares the photocatalytic CO_2_ reduction performances for all catalysts. The BMO/NO-C-7 catalyst exhibited superior CH_4_ and CO yields of 9.14 μmol g^−1^ h^−1^ and 14.49 μmol g^−1^ h^−1^, respectively.

To further verify the photocatalytic conversion of CH_4_ and CO products from CO_2_, ^13^CO_2_, and ^12^CO_2_ gas were employed for the ^13^C and ^12^C isotopic labeling. The GC-MS results are shown in Figure 7c. The fragment peaks of ^12^CH_4_ and ^13^CH_4_ appear at *m*/*z* = 16 and *m*/*z* = 17, while the main peaks of ^12^CO and ^13^CO appear at *m*/*z* = 28 and *m*/*z* = 29, confirming that CH_4_ and CO come from the photocatalytic CO_2_RR. To test the durability and stability of the BMO/NO-C-7 catalyst, we have carried out five cycles of photocatalytic CO_2_RR experiments, and Figure 7d clearly shows that the BMO/NO-C-7 catalyst maintained excellent stability after five cycles. Most remarkably, the yields of CH_4_ and CO are very stable.

### 3.3. Photocatalytic CO_2_ Reduction Mechanism

**Figure 8:**
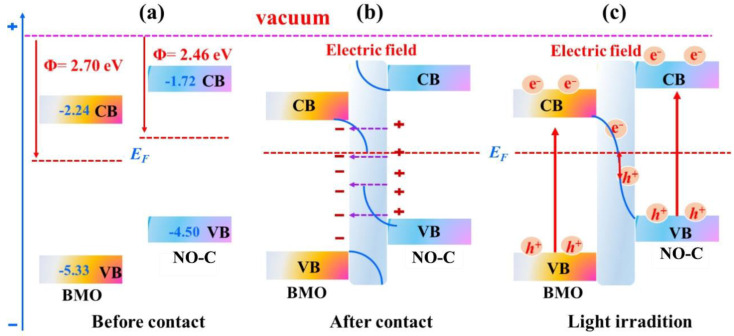
Diagram of the band edge positions before (**a**) and after contact (**b**) of BMO and NO-C. (**c**) The step-scheme electrons transfer diagram between BMO and NO-C under the full spectrum.

Combining the above experimental and theoretical results, Figure 8 summarizes the photocatalytic CO_2_ reduction mechanism of BMO/NO-C. The overall energy band diagrams of BMO and NO-C can be plotted based on the DFT calculations and experimental data. The Φ (Figure 8a) of BMO and NO-C are 2.70 and 2.46 eV, respectively, thus the *E_F_* level of BMO is lower than that of NO-C. When BMO and NO-C are in contact to form heterostructures, electrons migrate from NO-C to BMO until the *E_F_* reaches equilibrium (Figure 8b). The energy band of BMO bends downward due to electron accumulation. The energy band of NO-C bends upward. As a result, an internal potential field is formed from NO-C to BMO. Under the irradiation of light (Figure 8c), the electrons on either BMO or NO-C catalyst VB are excited to CB.

Meanwhile, due to the strong electrostatic force from the built-in electrical field formed by electron-transfer at the BMO/NO-C interfaces, the photoexcited electrons (e^−^) on the BMO CB migrate to the NO-C VB and combine with the holes (h^+^) left after photoexcitation. Therefore, the e^-^ on the BMO CB with weak reduction ability and the h^+^ on the NO-C VB with weak oxidation ability are consumed. As a result, the h^+^ on BMO VB undergoes an oxidation reaction, and the e^−^ on the NO-C CB undergoes a reduction reaction. The step-scheme transfer mechanism promotes the carrier separation and maintains the strong redox ability, which gives BMO/NO-C a strong driving force to participate in the photocatalytic CO_2_ reduction reaction.

## 4. Conclusions

We developed a simple method to synthesize the ultrathin 3D N,O-doped carbon networks, and fabricate the BMO/NO-C catalysts by using a solvothermal method. The prepared BMO/NO-C-7 photocatalyst has good photocatalytic CO_2_ reduction activity, and a long-term stability of 35 h with yields of 9.14 and 14.49 μmol g^−1^ h^−1^ for CH_4_ and CO, respectively. This excellent performance is attributed to the fact that the three-dimensional carbon skeleton does not collapse and stack and has a large specific surface area, which allows BMO nanoparticles to be uniformly distributed in NO-C. The resulting BMO/NO-C composites further promote chemical activity due to the synergistic effect and the formation of heterogeneous structures, where the transfer of electrons and the formation of holes under light irradiation provide channels for the transfer of gas molecules. This study presents an advanced 3D photocatalyst design strategy that has the potential to open up new avenues for photocatalysis.

## Data Availability

Not applicable.

## Supplementary Materials

The following supporting information can be downloaded at: https://www.mdpi.com/article/10.3390/nano13091569/s1, Figure S1: (a) Thermogravimetric (TG) and thermogravimetric derivative (DTG) curves for Cd^2+^-PVP at a heating rate of 10 °C min^−1^; Figure S2: N_2_ adsorption–desorption isotherms and pore size distributions of BMO/NO-C-5, BMO/NO-C-7, BMO/NO-C-9 and BMO; Figure S3: Models for (a) NO-C, (b) BMO (010) lattice plane and (c) Optimized BMO/NO-C structure model. (d) The differential charge density of the NO-C and BMO (010); Figure S4: (a,b) AFM image and height distribution curve of NO-C.
