# Supplementary material for: Bi2MoO6 Embedded in 3D Porous N,O-Doped Carbon Nanosheets for Photocatalytic CO2 Reduction
Source: Nanomaterials (Basel). 2023 May 6;13(9):1569. doi: 10.3390/nano13091569 (PMC10181000; doi:10.3390/nano13091569)
Supplement: Supplementary file 1 [file nanomaterials-13-01569-s001.zip › nanomaterials-2327022-supplementary.pdf]

Article

Bi_2MoO_6 Embedded in 3D Porous N,O-Doped Carbon Nanosheets for Photocatalytic CO_2 Reduction

Xue Bai [1,†], Lang He [1,†,*], Wenyuan Zhang [2], Fei Lv [1], Yayun Zheng [1], Xirui Kong [1], Du Wang [1,*] and Yan Zhao [1,*]

[1] The Institute of Technological Sciences, Wuhan University, Wuhan 430072, China

[2] School of Chemistry and Chemical Engineering, Harbin Institute of Technology, Harbin 150080, China

* Correspondence: langhe@whu.edu.cn (L.H.); wangdu@whu.edu.cn (D.W.); yan2000@whut.edu.cn (Y.Z.)
† These authors contributed equally to this work.

Materials

The reagents used in this experiment were of analytical purity and did not require further purification. Bismuth nitrate pentahydrate ($Bi(NO_3)_2 \cdot 5H_2O$) was purchased from Nanjing Reagent Chemical Co. Ltd, and Sodium molybdate dihydrate ($Na_2MoO_4 \cdot 2H_2O$) comes from Shanghai Reagent Wholesale Co. Ltd. Furthermore, ethylene glycol and ethanol comes from Wuhan Reagent Chemical Co. Ltd. Polyvinylpyrrolidone (PVP, MW = 1,300,000) comes from Jinan Reagent Wholesale Co. Ltd. Carbon dioxide gas ($^{12}CO_2$ 99.99 % and $^{13}CO_2$ 99.0 %), carbon monoxide gas (CO 99.90 %) and methane gas (CH_4 99.999 %) required for the test comes from Wuhan Wu Gang Gas Co. Ltd. Deionized (DI) water was used for all experiments.

Materials Characterization

The composition of the BMO, NO-C and BMO/NO-C nanocomposites was analyzed by X-ray diffraction (XRD, XPert Pro). The exploration of its microstructure and morphology was analyzed by SEM (JSM-IT800, 0.01–30 kV) and TEM (JEM-2100). X-ray photoelectron spectrometer (XPS) was employed to measure the surface chemical state of the photocatalyst. The Brunauer–Emmett–Teller (BET) specific surface area was determined by the N_2 adsorption-desorption method (JW-BK100B). Further analysis of the prepared material properties was also carried out using time-resolved photoluminescence (TR-PL) spectrum and fourier transform infrared (FT-IR) spectra.

Photocatalytic reduction of CO_2

We will use a custom Pyrex reactor (200 mL) to perform photocatalytic reduction experiments of CO_2 at RT. The light source is a 300 W xenon lamp (MICROSOLAR300) manufactured in China by Beijing Perfect Light Technology Co. Ltd. The sample was placed in the Pyrex reactor fixed sample table at the beginning of the experiment (sample preparation: 10 mg of sample was ultrasonically dispersed in 2.5 mL of DI water and 2.5 mL of ethanol for 3 min, and the suspension obtained was dried at 60 °C to obtain the target test sample), and the light source was located 10 cm directly in front of the sample, and 500 μL of DI water was also added to wet the dried sample before the test The sample is used as a proton source for photocatalytic CO_2RR. The equipment was cleaned with CO_2 gas at a rate of 30 mLmin^{-1} before testing, and a gas chromatograph (GC-9790) was used to analyze the photocatalytic products at a frequency of 30 min. The temperature of the system was kept constant at about ~26 °C by cooling the reaction device with water.

Photoelectrochemistry measurement

The electrochemical measurements were performed on an electrochemical workstation (CHI760C, Chinstruments, China) using a three-electrode system. A Pt foil was used as counter electrode, the Ag/AgCl electrode was used as reference electrode, and

the FTO conductive glass (1×1cm), whose conductive side was coated with thin sample film, was used as the working electrode. 0.5 M Na_2SO_4 solution was used as electrolyte.

DFT theoretical calculations

The work function (WF) of N-doped single-layer graphite carbon (NO-C) and BMO (010) slab have been calculated by using the CASTEP software. The generalized gradient approximation (GGA) with the Perdew-Burke Ernzerhof (PBE) functional is utilized to describe the exchange-correlation energy. The energy cutoff and Monkhorst-Pack k-point mesh are set to be 500 eV and 3 × 3 × 1, respectively. During the geometry optimization, the convergence tolerances are set as 1.0×10^{-5} eV/atom for energy and 0.03 eV/Å for maximum force. For the construction of surface models, a vacuum of 15 Å is used to eliminate interactions between periodic images. The work function is defined as $\Phi = E_V - E_F$, where E_V and E_F are the electrostatic potentials of the vacuum and Fermi levels, respectively.

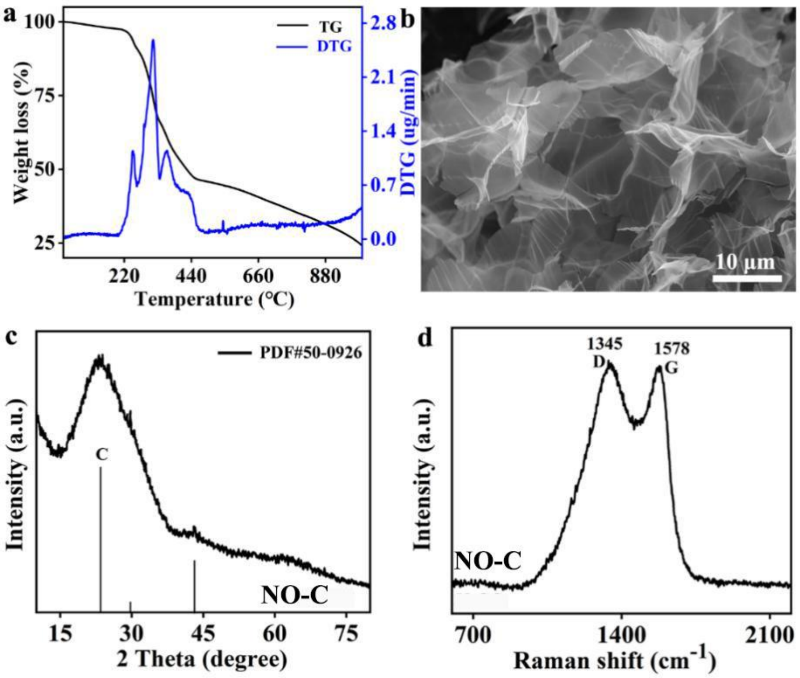

Figure S1 (a) Thermogravimetric (TG) and thermogravimetric derivative (DTG) curves for Cd^{2+}-PVP at a heating rate of 10 °C ·min^{-1}.

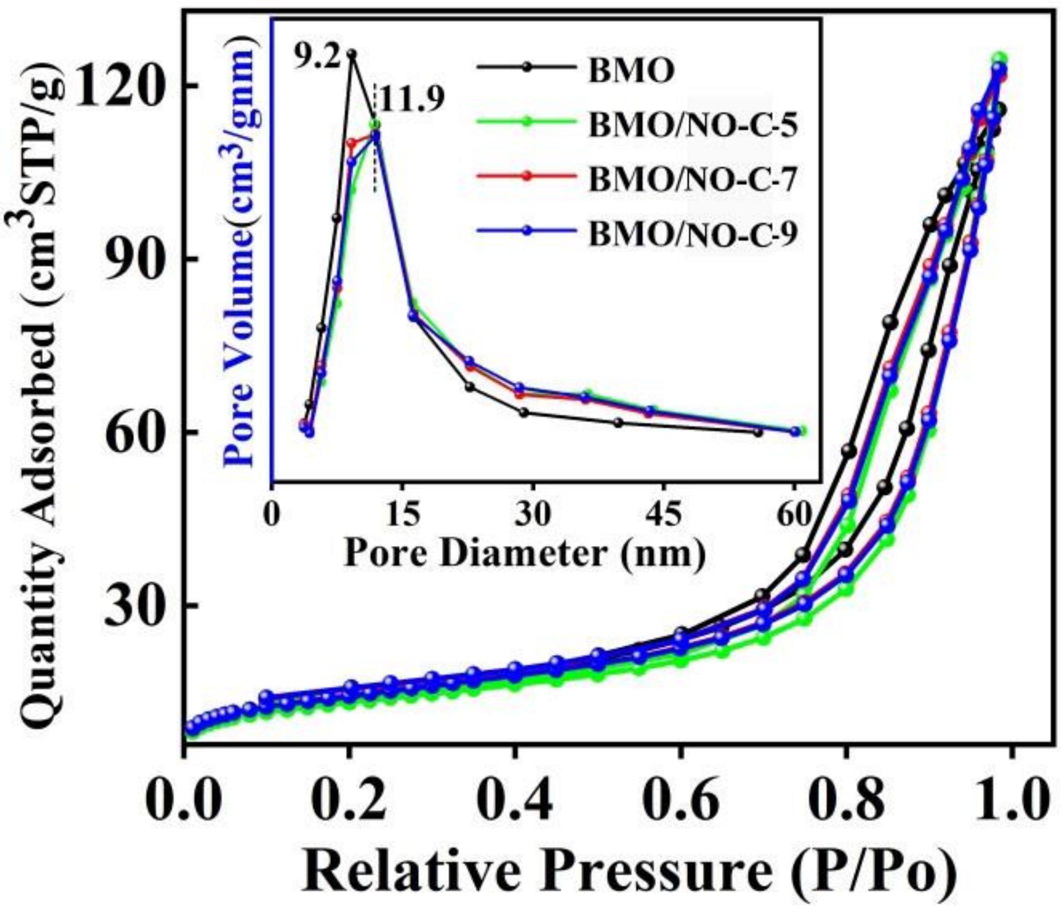

Figure S2 N_2 adsorption–desorption isotherms and pore size distributions of BMO/NO-C-5, BMO/NO-C-7, BMO/NO-C-9 and BMO.

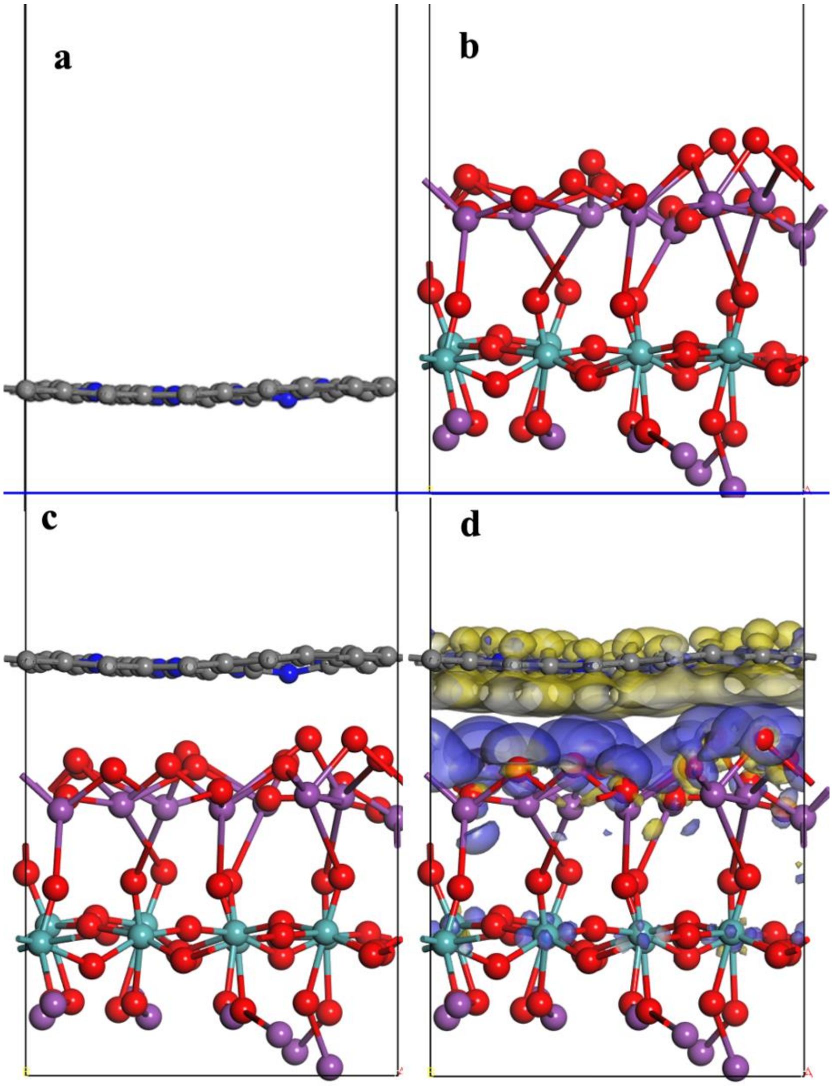

Figure S3 Models for (a) NO-C, (b) BMO (010) lattice plane and (c) Optimized BMO/NO-C structure model. (d)The differential charge density of the NO-C and BMO (010).

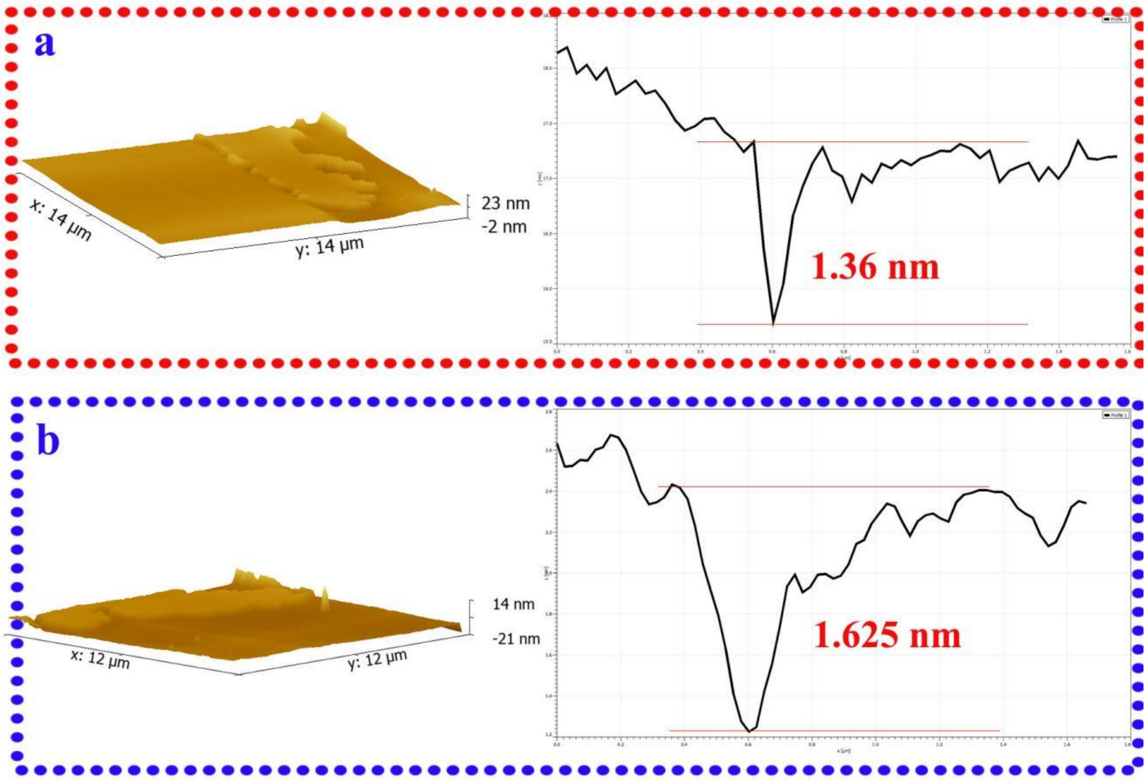

Figure S4 (a-b) AFM image and height distribution curve of NO-C.
